# Faster sequence homology searches by clustering subsequences

**DOI:** 10.1093/bioinformatics/btu780

**Published:** 2014-11-27

**Authors:** Shuji Suzuki, Masanori Kakuta, Takashi Ishida, Yutaka Akiyama

**Affiliations:** ^1^Graduate School of Information Science and Engineering, Tokyo Institute of Technology and ^2^Education Academy of Computational Life Sciences (ACLS), Tokyo Institute of Technology, Tokyo 152-8550, Japan

## Abstract

**Motivation:** Sequence homology searches are used in various fields. New sequencing technologies produce huge amounts of sequence data, which continuously increase the size of sequence databases. As a result, homology searches require large amounts of computational time, especially for metagenomic analysis.

**Results:** We developed a fast homology search method based on database subsequence clustering, and implemented it as GHOSTZ. This method clusters similar subsequences from a database to perform an efficient seed search and ungapped extension by reducing alignment candidates based on triangle inequality. The database subsequence clustering technique achieved an ∼2-fold increase in speed without a large decrease in search sensitivity. When we measured with metagenomic data, GHOSTZ is ∼2.2–2.8 times faster than RAPSearch and is ∼185–261 times faster than BLASTX.

**Availability and implementation:** The source code is freely available for download at http://www.bi.cs.titech.ac.jp/ghostz/

**Contact:** akiyama@cs.titech.ac.jp

**Supplementary information:** Supplementary data are available at *Bioinformatics* online.

## 1 Introduction

DNA sequencing technologies have improved rapidly. The HiSeq2500 can produce several hundred billion base pairs (bp) of sequence data in a single run and its throughput is **∼**10 000 times higher than that of old-generation sequencers. Most sequencers produce information in short fragments (reads) that range in length from 100 to 1000 bp. Thus, it is necessary to determine the location of each read in a genome, to use known biological information even when a reference genome is available. This process is called mapping, and many effective mapping programs, such as BWA (Li and Durbin, 2009, 2010) and Bowtie (Langmead and Salzberg, 2012; Langmead *et al.*, 2009), have been developed for this purpose.

In metagenomic analysis, environmental samples frequently include DNA sequences from many different species, and the reference database often does not contain closely related genome sequences. Thus, more sensitive approaches are required to identify novel genes in these samples. In a typical metagenomic analysis, reads are translated into protein coding sequences and assigned to protein families by running homology searches against publicly available databases, such as COG (Tatusov *et* *al.*, 1997, 2003) and Pfam (Finn *et* *al.*, 2010). The BLASTX program (Altschul *et* *al.*, 1990, 1997) is commonly used for such binning and classification searches. To identify homologs that may not have high nucleotide sequence identities, BLASTX translates nucleotide sequences into protein sequences, because protein sequences are often more similar than the original nucleotide sequences (Kurokawa *et al.*, 2007; Turnbaugh *et* *al.*, 2006). However, the search speed of BLASTX has become insufficient for analysis of the large quantities of sequence data now available.

Several currently available homology search tools are faster than BLAST, but possess decreased sensitivity. For example, BLAT (Kent, 2002) is **∼**50 times faster than BLAST; however, the search sensitivity of BLAT is much lower than that of BLAST and is often insufficient for metagenome sequence analysis. Thus, novel homology search tools, such as RAPSearch (Ye *et* *al.*, 2011), have recently been developed. RAPSearch has sufficient sensitivity for metagenomic analysis and a faster homology search speed than BLAST or BLAT because it uses a reduced amino acid alphabet (Murphy *et* *al.*, 2000) and a suffix array (Manber and Myers, 1993). In addition, RAPSearch2 has been improved to use hash tables instead of suffix arrays, making it more memory efficient (Zhao *et* *al.*, 2012).

However, several large metagenome projects, such as the Human Microbiome Project (HMP) (The Human Microbiome Project Consortium, 2012), the Metagenomics of the Human Intestinal Tract (MetaHIT) (Qin *et* *al.*, 2010) and the Earth Microbiome Project (Gilbert *et* *al.*, 2010), have recently produced unprecedentedly large amounts of sequence information. For instance, HMP has sequenced 681 human metagenome whole-genome shotgun samples. Therefore, there is a high demand for analysis of large amounts of metagenomic data. In addition, the numbers of reference sequences in databases will continue to increase with further improvements of sequencing technologies. For instance, the size of the National Center for Biotechnology Information (NCBI) non-redundant protein database (nr) increased from **∼**4.1 billion amino acid residues in 2010 to **∼**16.7 billion residues in 2014. Therefore, the speed of homology searches needs to be increased to facilitate metagenomic analysis.

To address the problem of increasing database size, CaBLASTP (Daniels *et* *al.*, 2013) introduced a compression approach and achieved a faster homology search than BLAST using the compressed database. CaBLASTP initially searches against a coarse database from which redundant subsequences have been removed, and then it uses these initial results to search the original database for similar sequences. This approach provides a more efficient homology search than BLAST, but the compression approach has proven to be difficult to apply to RAPSearch and BLAT, because their faster but less sensitive homology searches often fail to identify similar sequences in the compressed database, critically decreasing the sensitivity of the final search results.

To address some of these problems, we developed a new faster homology search method using database subsequence clustering. Current homology searches require a large amount of time to extend alignments without gaps, because the seed searches tend to produce large numbers of seeds (Vouzis and Sahinidis, 2011). However, only a small number of seeds produce ungapped extension scores that are higher than the score threshold, and the wasted computation time involved accounts for a large fraction of the time required for the ungapped extensions. Our method clusters subsequences from a database and filters out the non-representative seeds within these clusters to minimize the computation time spent on ungapped extensions. In our method, the subsequences in a cluster are more similar to representative subsequences than those obtained using CaBLASTP. Therefore, our method does not require high sensitivity in the initial search for representative subsequences in a cluster. In this research, we developed a novel fast homology search method that uses hash tables, and then applied our subsequence clustering technique to the index to further accelerate the sequence homology search algorithm. We implemented this algorithm as GHOSTZ.

## 2 Methods

### 2.1 Flow of the proposed homology search method using subsequence clustering

**Fig. 1.**
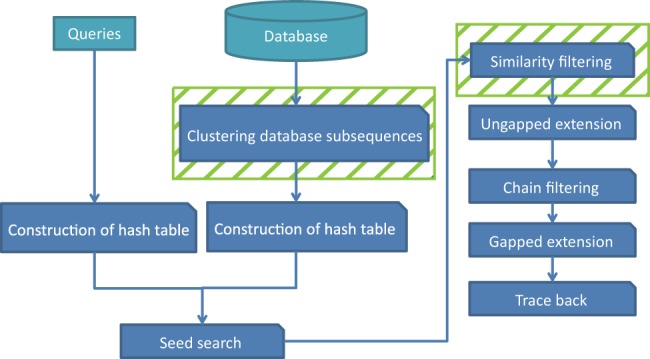
Flow of the proposed homology search method based on database subsequence clustering. Clustering of database subsequences and similarity filtering (shaded box) are included in this method

**Fig. 2.**
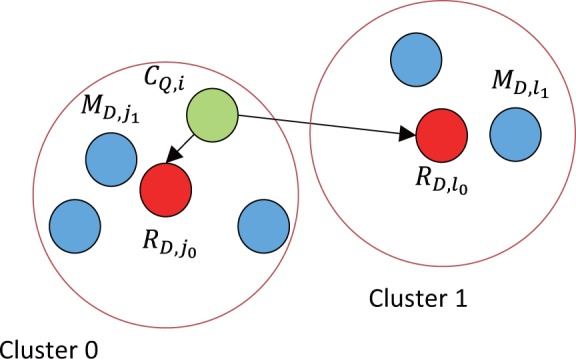
Example of similarity filtering. $C_{Q,i}$ is the query subsequence. $R_{D,j_{0}}$ and $R_{D,l_{0}}$ are the representative subsequences in the cluster 0 and the cluster 1, respectively. The lower bound of the distance between $C_{Q,i}$ and the member subsequence $M_{D,j_{1}}$ in the cluster 0 is calculated from the distance $d(C_{Q,i},R_{D,j_{0}})$. When the lower bound of $d(C_{Q,i},M_{D,j_{1}})\leq T_{\text{distance}}$, the seed for $C_{Q,i}$ and $M_{D,j_{1}}$ is taken into the next step. The lower bound of the distance between $C_{Q,i}$ and the member subsequence $M_{D,l_{1}}$ in the cluster 1 is calculated from distance $d(C_{Q,i},R_{D,l_{0}})$. When the lower bound of $d(C_{Q,i},M_{D,l_{1}})>T_{\text{distance}}$, the seed for $C_{Q,i}$ and $M_{D,l_{1}}$ is not taken into the next step

GHOSTZ adopts the seed-extension approach used in BLAST. The flow of GHOSTZ is shown in Figure 1. Subsequences are extracted from a database, and similar subsequences are clustered. Then, hash tables are constructed that contain indexes for the subsequences and the clusters. The homology search method uses the hash tables to select the seeds for the alignments from representative sequences in the clusters. The distance between a query subsequence and the cluster representative is calculated, and then the lower bounds of the distance between the query subsequence and other members of the cluster are computed based on triangle inequality, as shown in Figure 2. If the computed lower bound is lower than or equal to the distance threshold, the seed is taken into the next step, that is, ungapped extension, to investigate the homology between the query and the member sequences of the cluster. This filtering, using the lower bounds of the distance, is referred to herein as ‘similarity filtering’. Finally, chain filtering is used to bring similar extended seeds together, and a gapped extension is performed to obtain an alignment from the extended seed that contains gaps.

In the database subsequence clustering and seed search processes, the query and database amino acid sequences are both converted to a reduced amino acid alphabet to increase search sensitivity. We used a 10-letter reduced amino acid alphabet (A, {K, R}, {E, D, N, Q}, C, G, H, {I, L, V, M}, {F, Y, W}, P, {S, T}), which was derived based on the BLOSUM62 matrix (Murphy *et* *al.*, 2000). This reduced alphabet has been used successfully in previously reported research on homology searches (Ye *et* *al.*, 2011). For the ungapped and gapped extensions, the alignments are performed with the standard 20 amino acids.

### 2.2 Database subsequence clustering and construction of hash tables

**Fig. 3.**
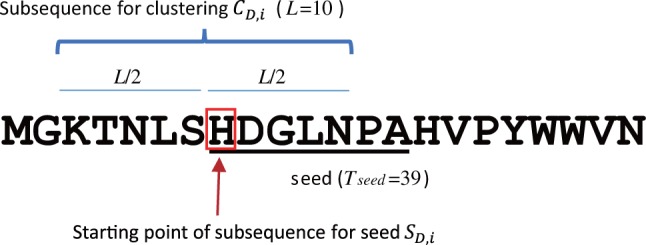
Relationship between a subsequence used for clustering and the starting position of the seed

The database subsequence clustering approach was developed for efficient homology sequence searches. In this method, subsequences in a database are clustered for use in similarity filtering; however, we do not cluster subsequences for seed themselves used in seed searches, but instead use longer subsequences, overlapping subsequences for seed (Fig. 3). To avoid confusion of terms, we use ‘subsequence for seed’ for subsequences used in general seed searches, denoted by *S*. And we use ‘subsequence for clustering’ for subsequences used in the database subsequence clustering and similarity filtering processes. These are denoted by *C*. All subsequences for clustering depend on a subsequence for seed. Therefore, GHOSTZ first builds a hash table of subsequences for seed, and then determines which subsequences should be used for clustering, using this hash table. Database subsequence clustering is performed using these subsequences. Therefore, we will first describe the construction of hash tables of subsequences for seed, and then describe the construction of subsequences for clustering and database subsequence clustering.

Here, the text $T=T[0,n]=t_{0}\ldots t_{n-1}$ denotes a sequence of symbols and the length of *T* is |T|=n. Each symbol is an element of an alphabet Σ (|Σ| of protein is 20). $T[i]=t_{i}$ and $T[i,i+j]=t_{i}\ldots t_{i+j-1}$ are substrings. The sequence of a query is *Q*. The sequences *D*_0_, *D*_1_, … ,$D_{N-1}$ in a database are connected by inserting delimiters to transform them into a single long sequence $D=\#D_{0}\#D_{1}\#,\ldots ,\#D_{N-1}\#$ (marked by the special symbol #). A seed is a pair of identical or similar subsequences of *Q* and *D*. $S_{Q,i}=Q[i,i+l]$ and $S_{D,j}=D[j,j+l]$ is the subsequence of *Q* and *D* for a seed, and $\left\{S_{Q_{i}},S_{D,j}\right\}$ is a seed. The hash table used to identify subsequences for seed stores the pair of hash values of $S_{D,i}$ and the starting point *i* of $S_{D,i}$.

In BLAST-like seed-extension algorithms, the search speed can be increased by decreasing the number of seeds. The number of seeds can be decreased if longer subsequences are used for seeds, because this decreases the number of randomly matched cases. However, this also causes a decrease in the search sensitivity. Thus, tolerances are required in the matching to retain sufficient search sensitivity. In BLASTX, the length of the subsequence for seed is three and neighborhood words are identified, as well as exact subsequences (Altschul *et* *al.*, 1997). A neighborhood word is a subsequence that is similar to each subsequence (Altschul *et* *al.*, 1990). BLAST uses a large variety of subsequences of each subsequence in a seed search to increase the search sensitivity using neighborhood words. However, neighborhood words are ineffective for longer subsequences for seed because the variety of neighborhood words is great.

GHOSTZ identifies long subsequences by employing a reduced amino acid alphabet in the seed search. The amino acid alphabet in the subsequences is converted to the reduced amino acid alphabet, and then the hash value for this subsequence is calculated. The variety of subsequences for each original subsequence becomes one, using the reduced amino acid alphabet. In addition, use of the reduced amino acid alphabet allows GHOSTZ to find longer subsequences without a large decrease in search sensitivity. In GHOSTZ, the length of the subsequence for seed is determined by the sum of the match scores of the subsequence. Because the frequency of each amino acid differs in the subsequences, the probability of finding each particular subsequence is different. Therefore, different subsequences may have different lengths. A score definition has previously been proposed for calculating matches between reduced amino acid alphabets (Melo and Marti-Renom, 2006). However, in this study we used a simpler definition. We defined the match scores of the groups of reduced amino acid alphabets by the largest match score in the group based on the original score matrix. For example, in the BLOSUM62 score matrix, the match scores of amino acids F, Y and W, are 6, 7 and 11, respectively; thus, the match score for the group including F, Y and W is 11. To avoid insignificant hits, only subsequences with scores that exceed the score threshold *T*_seed_ are hashed as subsequences for seed. For example, when *T*_seed_ = 39, ‘HDGLNP’ is not used in the seed search because its score is 38 and does not exceed *T*_seed_. However, ‘HDGLNPA’ is used in the seed search because its score is 42, which exceeds *T*_seed_. In addition, in our implementation, the length of subsequences for seed is restricted to 6–8 residues, because a perfect hash function is used.

**Fig. 4.**
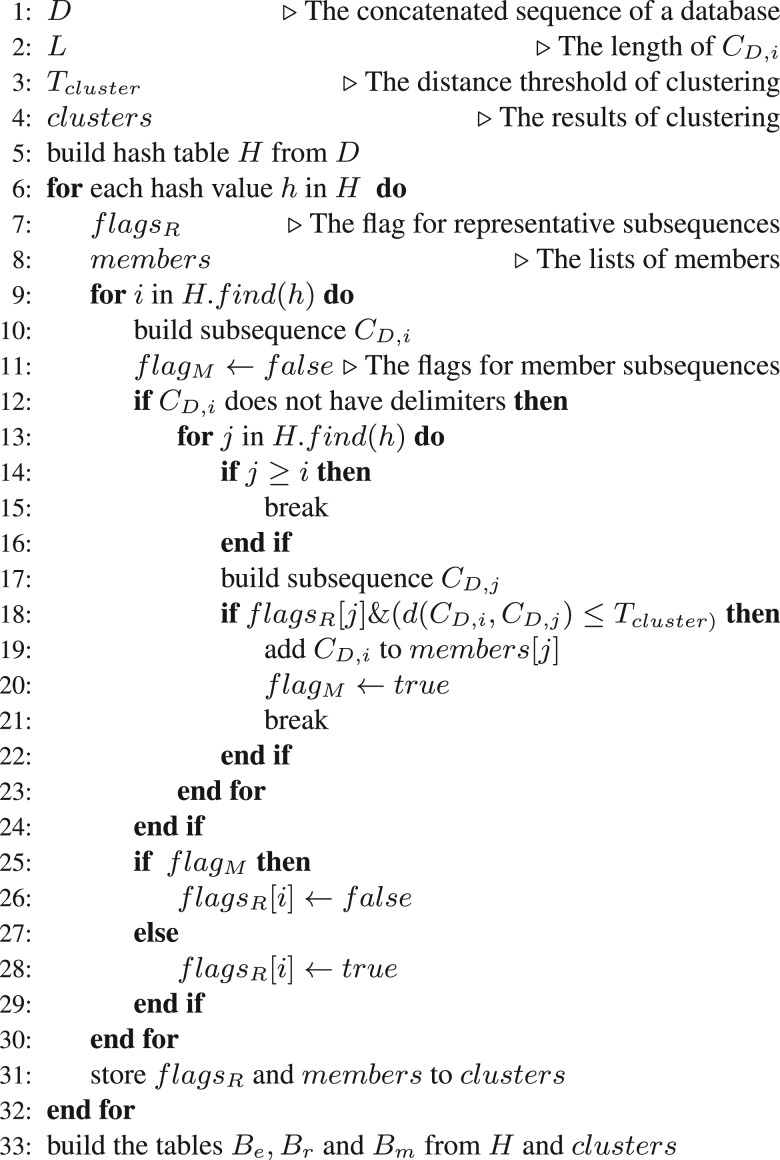
Pseudo-code for database subsequence clustering

After building the hash table of subsequences for seed, the subsequences for clustering are constructed, and database subsequence clustering is performed as follows: If *i* is the starting point of $S_{D,i}=D[i,j]$ and *L* is the length of the subsequence used for clustering, then let $C_{D,i}=D[i-L/2,i+L/2]$ be the subsequence for clustering. For clustering, a subsequence for clustering with *i* as the center is used, instead of a subsequence for seed with *i* as the starting point. The relationship between $C_{D,i}$ and $S_{D,i}$ is shown in Figure 3. If $C_{D,i}$ has delimiters, $C_{D,i}$ are not used for clustering because $C_{D,i}$ contains the subsequence of several sequences in the database. $C_{D,i}$ becomes a member of a cluster if it has the same hash value of $S_{D,j}$ as the cluster representative $C_{D,j}$ and the distance between the representative of a cluster $C_{D,j}$ and $C_{D,i}$ is lower than or equal to the distance threshold *T*_cluster_. Hamming distance, which is the number of mismatches between sequences, is used to measure this distance. To reduce the computation time required for clustering, a greedy algorithm similar to CD-HIT (Fu *et al.*, 2012; Li and Godzik, 2006) was employed. The algorithm for database subsequence clustering is shown in Figure 4. In this algorithm, the first subsequence sampled always becomes a cluster representative. All subsequences are compared with each cluster representative, and the subsequence becomes a new cluster representative if it is not a member of any other cluster. Before running the database subsequence clustering, we recommend that similar sequences are arranged close to each other in the input file, using a clustering tool such as CD-HIT, because this allows the clustering algorithm to cluster subsequences more efficiently. After subsequence clustering, the results are used to construct three tables to be used as indexes for the seed searches. The *B_e_* hash table stores the hash values of $S_{D,i}$ and the starting points *i* of $S_{D,i}$ for the representatives of clusters where the number of members in the cluster is only one. The *B_r_* hash table stores the hash values of $S_{D,i}$, their cluster IDs and the starting points *i* of $S_{D,i}$ that are representative of a cluster (not stored in *B_e_*). The *B_m_* table stores the mapping from the cluster IDs to the starting points *i* of $S_{D,i}$ whose $C_{D,i}$ are members of that cluster. These three tables are used for the seed search. Examples of *B_e_*, *B_r_* and *B_m_* are shown in Supplementary Figure S1A–C.

### 2.3 Seed search and similarity filtering

The seed search is performed with *B_e_*, *B_r_*, *B_m_* and the hash table of queries. The hash table of the queries is constructed before the seed search. This hash table contains the hash values of $S_{Q,i}$, the query IDs and the starting points of subsequences for the corresponding hash values. An example of a hash table of queries is shown in Supplementary Figure S1D.

In the seed search, seeds of query subsequences and representative subsequences in the database are found using *B_e_* and *B_r_*. If the seeds are from *B_e_*, an ungapped extension is performed because there are no other subsequences in the cluster. If the seeds are from *B_r_*, the similarity filtering process is performed. Then, the hamming distance between a query and the database subsequence is calculated. Given two sequences *S*_1_ and *S*_2_, we denote by $d(S_{1},S_{2})$ the distance between *S*_1_ and *S*_2_. The distance should satisfy the following triangle inequality:

(1) $$d(S_{1},S_{2})\leq d(S_{1},S_{3})+d(S_{2},S_{3})$$

If $C_{Q,i}$ is the subsequence of the query, $M_{D,j}$ ($C_{D,j}$) is the sequence of a cluster member, and $R_{D,k}$ ($C_{D,k}$) is the subsequence of a representative cluster member, then the lower bound of the distance between $R_{D,i}$ and $M_{D,j}$ from this inequality will be:

(2) $$d(C_{Q,i},M_{D,j})\geq d(C_{Q,i},R_{D,k})-d(R_{D,k},M_{D,j})$$

**Fig. 5.**
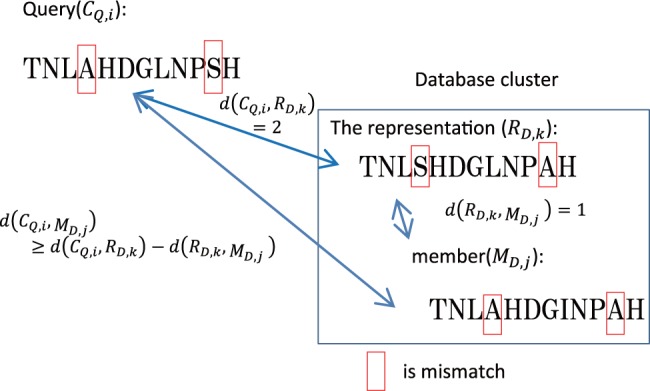
Relationships among a query subsequence, a representative cluster subsequence and a member of the cluster that satisfies the triangle inequality

**Fig. 6.**
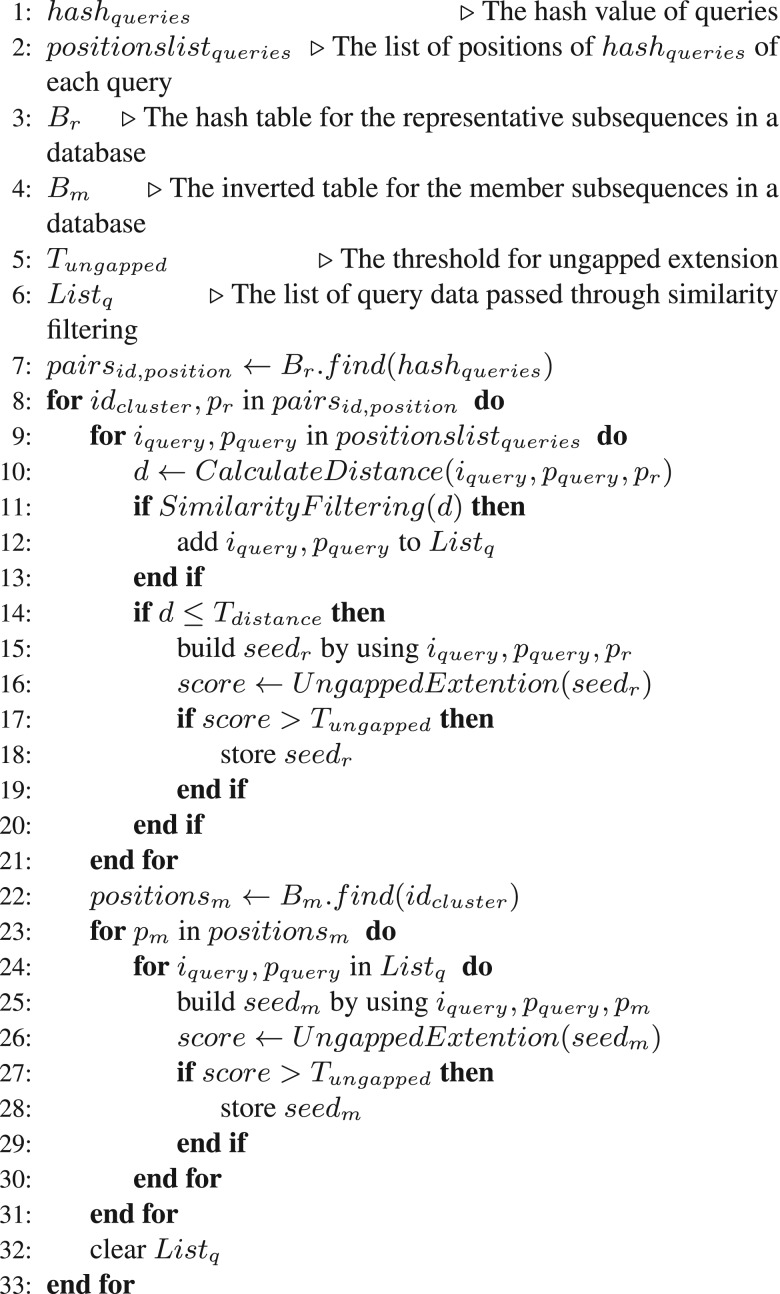
Pseudo-code for seed search, similarity filtering and ungapped extension in the case of multiple cluster members

This lower bound of the distance between $C_{Q,i}$ and $M_{D,j}$ is calculated, and the seed is extended without gaps if this lower bound of the distance is less than or equal to the distance threshold *T*_distance_. The relationships among the query, the cluster representative and the cluster members are shown in Figure 5. The pseudo-code for the seed search and similarity filtering is shown in Figure 6.

### 2.4 Ungapped extension

Gapped extension generally requires large amounts of computation time; therefore, most homology search algorithms perform an ungapped extension before a gapped extension. We used an ungapped extension to filter candidate seeds in the output from the seed search. Only seeds with ungapped extension scores that exceed the score threshold *T*_ungapped_ are stored and extended with gaps after the ungapped extension is complete. In the ungapped extension, the cutoff technique that is used in BLAST (Altschul *et* *al.*, 1990) is used to accelerate the extension process. The *T*_ungapped_ and the other parameters for ungapped extensions are the same as the BLAST default parameters.

To access memory efficiently when performing the ungapped extension, seed searches are performed for multiple queries simultaneously. If the hash values of query subsequences are the same, their starting points are packed using the hash table. Then, an ungapped extension is performed for the queries that have identical hash values in sequential order, because this increases the cache hit ratio when accessing the positions of the sequences in the database (line 9–31 in Fig. 6).

### 2.5 Chain filtering and gapped extension

Chain filtering is performed after an ungapped extension because some seeds overlap. Therefore, the number of gapped extensions can be reduced by merging overlapping seeds. After chain filtering, the seeds are extended with gaps using a score-limited dynamic programming technique (Altschul *et* *al.*, 1997).

### 2.6 Execution of the homology search method without subsequence clustering

**Fig. 7.**
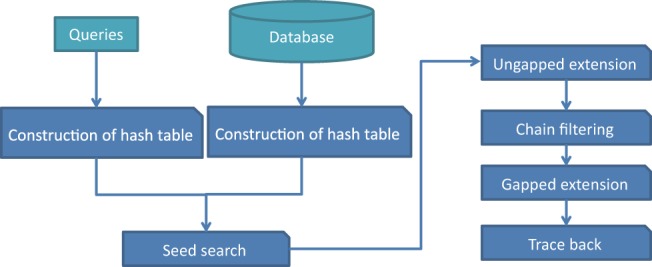
Flow of the proposed homology search method without database subsequence clustering for the purpose of comparison

The flow of the homology search without subsequence clustering is shown in Figure 7. This method is almost identical to that used in GHOSTZ, except that subsequence clustering and similarity filtering are not used for the seed search. This method, without subsequence clustering, was used to evaluate the improvement in processing time obtained by subsequence clustering. Here, the query subsequences are searched against all the subsequences in the database using hash tables. Next, all seeds are directly extended using the ungapped extension process. Finally, chain filtering is performed to merge similar seeds, and gapped extension is used to extend the seed sequences.

## 3 Results

### 3.1 Datasets and the computing environment

We evaluated the performance of the homology searches with and without subsequence clustering. We used the amino acid sequences in the Kyoto Encyclopedia of Genes and Genomes (KEGG) GENES database (as of May 2013). This database contains **∼**10 million protein sequences, which comprise a total of **∼**3.6 billion residues. For the query sequences, we used three datasets: soil microbiome metagenomic sequences (accession number, SRR407548, read length = 150 bp), human microbiome metagenomic sequences (accession number, SRS011098, read length = 101 bp) and marine microbiome metagenomic sequences (accession number, ERR315856, read length = 104 bp). SRR407548 and ERR315856 were obtained from the DNA Data Bank of Japan Sequence Read Archive, which is a member of the International Nucleotide Sequence Database Collaboration and which archives data in close collaboration with the NCBI Sequence Read Archive and the European Bioinformatics Institute Sequence Read Archive. SRS011098 was obtained from the Data Analysis and Coordination Center for the HMP web site (http://www.hmpdacc.org/). We used the whole metagenomic shotgun sequencing data from SRS011098. For all datasets, 10 000 DNA short reads were randomly selected and used. The evaluation tests were performed on a workstation with a 2.93 GHz Intel Xeon 5670 processor, 54 GB memory and SUSE Linux Enterprise Server 11 Service Pack (SP) 1.

For the homology search with and without subsequence clustering, we used a seed score threshold of *T*_seed_ = 39. *T*_seed_ was determined to be similar in sensitivity to RAPSearch. The parameters used for gapped and ungapped extensions were the same as the BLASTX default parameters. To perform the database subsequence clustering efficiently, similar sequences were arranged close to each other in the database file, based on the results of CD-HIT.

### 3.2 Relationship between subsequence length and acceleration ratio and accuracy

The subsequence clustering method has three parameters: the length of the subsequence *L*, the distance threshold for the representative of a cluster *T*_cluster_ and the distance threshold for the similarity check *T*_distance_. The subsequence length *L* particularly affects the performance of the search method because *T*_cluster_ and *T*_distance_ depend on *L*; therefore, we first determined the optimal length of a subsequence using *L* = 6, 8, 10, 12 and 14 and fixed distance thresholds of *T*_cluster_ = 0.1 *L* and *T*_distance_ = 0.2 *L*. We used 10 000 randomly selected DNA short reads from soil microbiome metagenomic sequences (SRR407548) and the KEGG GENES database. The acceleration ratios with different *L* for the subsequence clustering search method over the method without subsequence clustering are shown in Table 1. As shown, the speed of the search method that included subsequence clustering increased when *L* decreased.

**Table 1.** Computation times for homology searches using different subsequence lengths for the SRR407548 reads against the KEGG GENES database

	Computation time (s)	Acceleration ratio
Without clustering	936.5	1.0
*L* = 6	348.7	2.7
*L* = 8	384.3	2.4
*L* = 10	460.8	2.0
*L* = 12	460.8	2.0
*L* = 14	509.3	1.8

*Note*: *L* is the length of the subsequence. The acceleration in processing speed is given as the ratio of the time used for the search method with subsequence clustering relative to the time used for the search method without subsequence clustering.

**Fig. 8.**
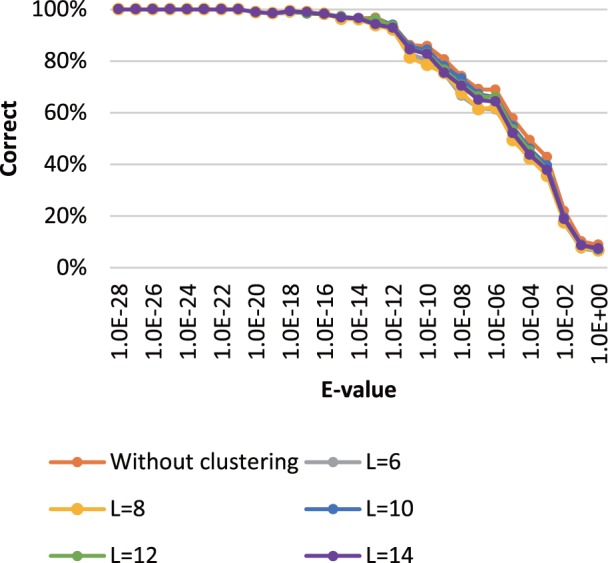
Search accuracy of GHOSTZ for the SRR407548 sequence alignments against the KEGG GENES database. The percentage of correct answers is shown on the vertical axis. The *E*-values of the alignments are shown on the horizontal axis

The accuracy of the homology search for the different query sequences was estimated using the search results obtained by the Smith–Waterman local alignment algorithm implemented in SSEARCH (Pearson, 1991) as the correct result. The performance was estimated in terms of the fraction of the results that corresponded to the correct result. A search result was considered to be correct when the subject sequence with the highest score in SSEARCH was the same as the subject sequence obtained by our search method with subsequence clustering. The accuracy of *L* = 10 was better than the accuracy of the other lengths (Fig. 8). Therefore, we determined that *L* = 10 was the optimal subsequence length because it yielded a good balance between sensitivity and computation time. GHOSTZ, using database subsequence clustering, achieved an **∼**2-fold increase in processing speed, without a large decrease in search accuracy.

### 3.3 Comparison of the proposed search method with other methods

To further evaluate GHOSTZ, we compared its search accuracy and computation time with the accuracy and computation time of NCBI BLAST (version 2.2.28+), BLAT (version 34 standalone) and RAPSearch (version 2.12). The metagenomic DNA sequences (SRR407548, SRS011098 and ERR315856) and the KEGG GENES database were used to evaluate these tools. The BLASTX program was executed with the command line options ‘-outfmt 6 -comp_based_stats 0’, which instructed the program to output in tabular format. Composition-based statistics (Altschul *et* *al.*, 2005) were not used because this method was not employed in SSEARCH. The BLAT program does not include a function to translate the DNA reads to protein sequences; therefore, we translated the DNA reads into protein sequences based on the standard codon table. The BLAT program was executed with the command line options ‘-q = prot -t = prot -out = blast8’, which instructed the program to use protein queries and a protein database, and to output the data in the BLAST tabular format. The RAPSearch program was executed with the default command line options. The proposed homology search method was executed with *L* = 10.

**Fig. 9.**
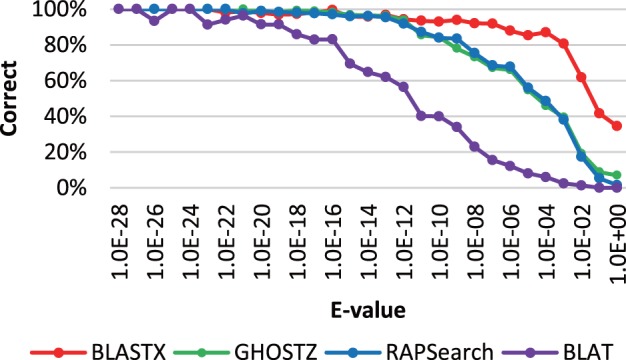
Search accuracy of different search methods for the SRR407548 sequence alignments against the KEGG GENES database. The percentage of correct answers is shown on the vertical axis. The *E*-values of the alignments are shown on the horizontal axis

The accuracy was evaluated in the same way as the relationship between the length of the subsequence and the acceleration ratio. The results for SRR407548, SRS011098 and ERR315856 are shown in Figure 9, Supplementary Figures S2 and S3, respectively. The accuracy of GHOSTZ was better than that of BLAT and was almost equal to that of RAPSearch. However, the accuracy of GHOSTZ was lower than that of BLASTX, especially for hits with *E*-values above $1.0\times E^{-6}$. However, hits with such high *E*-values are not used in practice because they are unreliable. For instance, Turnbaugh *et al.* (2006) used hits with *E*-values below $1.0\times E^{-5}$, and Kurokawa *et al.* (2007) used hits with *E*-values below $1.0\times E^{-8}$. Therefore, we consider that GHOSTZ has sufficient search accuracy for most metagenomic applications.

The computation time of each method was also evaluated. The software was run with the same commands that were used to evaluate the search accuracy. The computation times of the tested methods for SRR407548, SRS011098 and ERR315856 are shown in Table 2, Supplementary Tables S1 and S2, respectively. GHOSTZ had the fastest search speed of the software packages tested. GHOSTZ achieved **∼**185.2–261.3 times faster processing than BLASTX, and **∼**2.2–2.8 times faster processing than RAPSearch.

**Table 2.** Computation times for the SRR407548 reads against the KEGG GENES database

	Computation time (s)	Acceleration ratio
GHOSTZ	460.8	261.3
RAPSearch	1285.5	93.7
BLAT	2514.9	47.9
BLASTX	120395.2	1.0

*Note*: The acceleration in processing speed for the search method using subsequence clustering relative to BLASTX using one thread.

We also measured the search accuracy and computation time of each tool using different parameters. We used 10 000 randomly selected DNA short reads from SRR407548 and the KEGG GENES database. Because it is difficult to compare many plots showing the results for various parameters, we used single-value accuracy, which is calculated as the ratio of correctly searched queries to all queries whose *E*-values $<1.0\times E^{-3}$. These evaluations were performed on the same workstation used for the other evaluation, but the version of the operating system had been updated from SUSE Linux Enterprise Server 11 SP1 to SP3. Using this computing environment and these measurements, the computation time of GHOSTZ was 396.3 s and the accuracy was 0.84. Supplementary Tables S3, S4 and S5 show the accuracy and computation time of RAPSearch, BLAT and BLASTX, respectively, using different parameters. RAPSearch showed a drastic decrease in accuracy in the fast mode, and the accuracy of BLAT was not drastically improved the accuracy, even using a smaller tile size parameter. Using the fastest parameter, the accuracy of BLASTX was similar to that of GHOSTZ; however, the computation time required was much greater.

### 3.4 Evaluation of memory size

The amount of memory required for running GHOSTZ depends on the size of database. Current computing systems often have relatively small memory sizes relative to the size of current databases. Therefore, GHOSTZ divides a database into several chunks. GHOSTZ sequentially searches each database chunk, and merges its results with the results of previous chunk searches, when this chunk division is performed before the construction of its database indexes. The default chunk size is 1 GB. Using this approach, GHOSTZ dramatically reduces working memory requirements. However, even using this technique, GHOSTZ requires more memory than RAPSearch. When we used 10 000 randomly selected DNA short reads from soil microbiome metagenomic sequences (SRR407548) and the KEGG GENES database, GHOSTZ required **∼**41 GB of memory for constructing the indexes of the database, and **∼**7 GB for the homology search itself (Table 3). In contrast, RAPSearch required only **∼**4 GB for the homology search. However, GHOSTZ can reduce the memory required by decreasing the database chunk size. As shown in Table 3, the memory required for GHOSTZ increases nearly linearly in proportion to the size of the database chunks. If a database is divided into a larger number of chunks, the memory required decreases accordingly. Of course, a trade-off exists between database chunk size and search speed. Homology search computation times increase as the size of a database chunk decreases. This is so because the number of clusters increases and the cache hit ratio in ungapped extension decreases. However, the situation is not dire, as shown in Table 4, the search speed of GHOSTZ with 128 MB chunks is **∼**12% slower than that with 1 GB chunks. Therefore, using smaller database chunks, GHOSTZ is executable even on a typical PC.

**Table 3.** Memory usage for database construction and homology search with various database chunk sizes

Tool (chunk size)	Memory size for constructing index (GB)	Memory size for homology search (GB)
GHOSTZ (128 MB)	5.4	1.4
GHOSTZ (256 MB)	10.1	2.2
GHOSTZ (512 MB)	21.0	3.8
GHOSTZ (1 GB)	41.0	6.7
RAPSearch	6.9	4.1

*Note*: The first, second and third columns show the size of the database chunks, the memory required for constructing the index (GB) and the memory required for the homology search (GB), respectively. We searched the KEGG GENES (3.9 GB) database.

**Table 4.** Computation time for database construction and homology search with various database chunk sizes

Tool (chunk size)	Computation time (s)	Acceleration ratio
GHOSTZ (128 MB)	545.2	0.88
GHOSTZ (256 MB)	488.2	0.94
GHOSTZ (512 MB)	479.1	0.96
GHOSTZ (1 GB)	460.8	1.00
RAPSearch	1285.5	0.35

*Note*: The first, second and third columns show the size of the database chunks, the computation time and the acceleration in processing speed relative to GHOSTZ, respectively, using 1 GB database chunks. We searched the KEGG GENES (3.9 GB) database.

## 4 Discussion

In the evaluation experiment, GHOSTZ achieved an **∼**2-fold increase in speed, relative to GHOSTZ without clustering. This acceleration can probably be attributed to the reduction in the number of ungapped extensions that were required when using GHOSTZ. To validate this hypothesis, we compared the total number of ungapped extensions required by each method. In the database subsequence clustering approach, similarity filtering requires comparable computing time to the ungapped extension process; therefore, we added the number of similarity filterings performed to the number of ungapped extensions. We found that the number of ungapped extensions could be reduced to approximately one-third of the original number using database subsequence clustering. Currently, ungapped extension is one of the primary bottlenecks in fast homology searches. Thus, we think that this effective decrease in ungapped extensions contributed to the large acceleration we observed when using subsequence clustering for homology searches.

CaBLASTP, which is based on a compression approach, achieved 2.4–3.1-fold faster processing speed than the original BLASTP (Daniels *et* *al.*, 2013). The acceleration achieved by GHOSTZ was **∼**2-fold faster than the speed of GHOSTZ without clustering, which is comparable to that of CaBLASTP. However, we achieved a processing speed that was much faster than that of the BLAST homology search algorithm. As described earlier, the compression approach used in CaBLASTP requires high search sensitivity and cannot be applied to faster, but less sensitive, homology search algorithms. In the initial processing, the compression search algorithm needs to find remote homologs in a coarse database, and less sensitive homology search algorithms often fail to find such sequences. In contrast, the clustering targets used in our approach are subsequences in a database, which does not depend on search sensitivity. In addition, GHOSTZ uses hamming distance in database subsequence clustering to measure the dissimilarity between sequences, whereas CaBLASTP uses sequence similarity. Using distance allows search seeds to be pruned efficiently using triangle inequality in an ungapped extension process, contributing to the acceleration in processing.

GHOSTZ allows the indexes of a database to be constructed anew, so that users who wish to use other parameters can use this method. Construction of database indexes for a 1 GB database requires **∼**3 h of computation time. However, when a huge number of DNA reads obtained using next-generation sequencing are to be processed, the computation time for homology searches is generally much greater than the time required for database construction. Therefore, we consider that the computational time involved in rebuilding database indexes and clustering is not likely to be a problem in practice.

### 4.1 Evaluation of computing time for more queries

Reading of database files, including indexes, accounts for a larger fraction of the computing time required for GHOSTZ, compared with the other tools evaluated, especially if the number of queries is small. In Section 3, we used 10 000 queries for evaluation because of the limitations imposed by our computational resources. Thus, the performance of GHOSTZ might have been underestimated. To investigate the point, we evaluated the computation time required for GHOSTZ and RAPSearch for 1000, 10 000, 100 000 and 1 000 000 queries. The queries were randomly selected from SRR407548 and searched against the KEGG GENES database. These evaluation tests were performed on a workstation with a 2.93 GHz Intel Xeon 5670 processor, 54 GB memory and SUSE Linux Enterprise Server 11 Service Pack 3. The acceleration ratio of GHOSTZ relative to RAPSearch is shown in Supplementary Figure S4. When 1000 queries were used, the acceleration was only 2.9. In contrast, GHOSTZ was **∼**3.8-fold faster than RAPSearch when the number of queries was 1 000 000. These results indicate that GHOSTZ achieves its full potential only when working on a sufficient number of queries.

## 5 Conclusion

We developed a new homology search algorithm with subsequence clustering. We reduced the number of ungapped alignment extensions by clustering subsequences in a database, and achieved a 2-fold acceleration in processing speed without a drop in search sensitivity. The algorithm was designed for functional and taxonomic annotation in metagenome analysis. The proposed database subsequence clustering method could also be useful in proteome research, which requires a huge number of sequence homology searches.

## Supplementary Material

Supplementary Data
