# Supplementary material for: Faster sequence homology searches by clustering subsequences
Source: Bioinformatics. 2014 Nov 27;31(8):1183–90. doi: 10.1093/bioinformatics/btu780 (PMC4393512; doi:10.1093/bioinformatics/btu780)
Supplement: Supplementary Data [file supp_31_8_1183__index.html]

Faster sequence homology searches by clustering subsequences — Faster sequence homology searches by clustering subsequences — Supplementary Data 

# Faster sequence homology searches by clustering subsequences

## Supplementary Data

files

**Files in this Data Supplement:**

- Supplementary Data - doc file
