# Supplementary material for: Faster sequence homology searches by clustering subsequences
Source: Bioinformatics. 2014 Nov 27;31(8):1183–90. doi: 10.1093/bioinformatics/btu780 (PMC4393512; doi:10.1093/bioinformatics/btu780)
Supplement: Supplementary Data [file supp_btu780_SupplementaryData.doc]

**
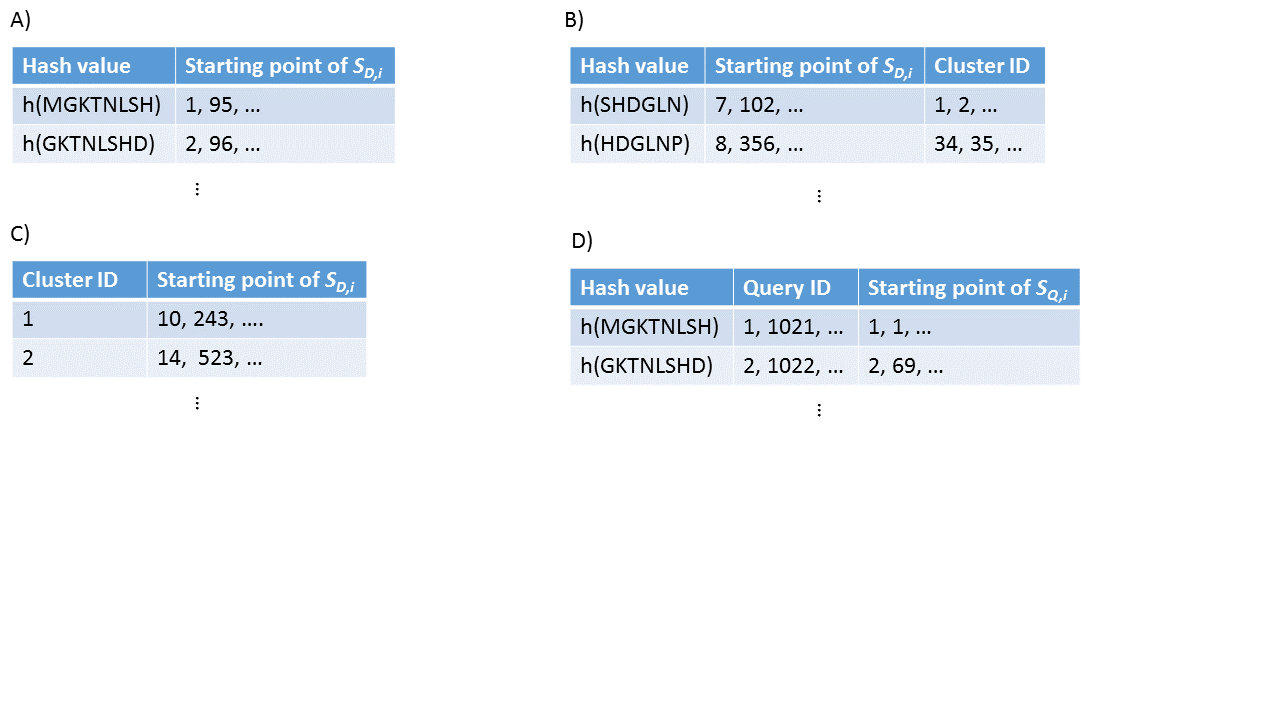
**

**Figure S1.** Examples of data structures. A) An example of a hash table *B_e_*. *B_e_* stores the hash values of *S_D,i_* and the starting points *i* of *S_D,i_* that are the representatives *C_D,i_* of clusters where the number of members in the cluster is only one. B) An example of a hash table *B_r_*. *B_r_* stores the hash values of *S_D,i_*, their cluster IDs, and the starting points *i* of *S_D,i_* that are the representative *C_D,i_* of a cluster (not stored in *B_e_*). C) An example of a table *B_m_*. *B_m_* that stores the mapping from the cluster IDs to the starting points *i* of *S_D,i_* whose *C_D,i_* are members of that cluster. D) An example of a hash table of the queries. This hash table contains the hash values of *S_Q,i_*, the query IDs, and the starting points *i* of subsequences for the corresponding hash values.

**Figure S2.** Search accuracy of different search methods for the SRS011098 sequence alignments against the KEGG GENES database. The percentage of correct answers is shown on the vertical axis. The E-values of the alignments are shown on the horizontal axis.

**Figure S3.** Search accuracy of different search methods for the ERR315856 sequence alignments against the KEGG GENES database. The percentage of correct answers is shown on the vertical axis. The E-values of the alignments are shown on the horizontal axis.

**Figure S4.** Acceleration ratio of GHOSTZ to RAPSearch for 1,000, 10,000, 100,000, and 1,000,000 queries. The number of queries is shown on the horizontal axis. The acceleration ratio is shown on the vertical axis.

**Table S1.** Computation times for the SRS011098 reads against the KEGG GENES database

|  | Computation time (sec.) | Acceleration ratio |
| --- | --- | --- |
| GHOSTZ | 298.6 | 205.5 |
| RAPsearch | 691.1 | 88.8 |
| BLAT | 1251.5 | 49.0 |
| BLASTX | 61371.0 | 1.0 |

The acceleration in processing speed relative to BLASTX using one thread.

**Table S2.** Computation times for the ERR315856 reads against the KEGG GENES database

|  | Computation time (sec.) | Acceleration ratio |
| --- | --- | --- |
| GHOSTZ | 388.2 | 185.2 |
| RAPsearch | 870.4 | 82.6 |
| BLAT | 1619.7 | 44.4 |
| BLASTX | 71900.8 | 1.0 |

The acceleration in processing speed relative to BLASTX using one thread.

**Table S3.** Relationship between RAPSearch parameters and performance

| RAPSearch parameters | Accuracy | Computation time (sec.) |
| --- | --- | --- |
| -a F (default) | 0.84 | 1469.5 |
| -a T | 0.54 | 156.3 |

We changed -a F (default) and T, which instructed the program to perform a fast mode search. The first, second, and third columns show the parameters, the accuracy, and the computation time.

**Table S4.** Relationship between BLAT parameters and performance

| BLAT parameters | Accuracy | Computation time (sec.) |
| --- | --- | --- |
| -tileSize 4 | 0.48 | 65979.0 |
| -tileSize 5 (default) | 0.40 | 2818.0 |
| -tileSize 6 | 0.35 | 492.9 |

We changed –tileSize, which is the subsequence length for the seed search, to 4, 5 (default), and 6. The first, second, and third columns show the parameters, the accuracy, and the computation time.

**Table S5.** Relationship between BLASTX parameters and performance

| BLASTX parameters | Accuracy | Computation time (sec.) |
| --- | --- | --- |
| -threshold 12 -word_size 3 (default) | 0.93 | 113517.3 |
| -threshold 12 -word_size 4 | 0.93 | 182071.8 |
| -threshold 12 -word_size 5 | 0.93 | 298595.0 |
| -threshold 12 -word_size 6 | N/A | N/A |
| -threshold 12 -word_size 7 | N/A | N/A |
| -threshold 14 -word_size 3 | 0.92 | 41032.6 |
| -threshold 14 -word_size 4 | 0.93 | 65636.3 |
| -threshold 14 -word_size 5 | 0.93 | 126303.4 |
| -threshold 14 -word_size 6 | 0.93 | 264059.3 |
| -threshold 14 -word_size 7 | 0.93 | 217667.0 |
| -threshold 16 -word_size 3 | 0.91 | 26589.0 |
| -threshold 16 -word_size 4 | 0.92 | 27609.1 |
| -threshold 16 -word_size 5 | 0.93 | 58431.0 |
| -threshold 16 -word_size 6 | 0.93 | 109505.2 |
| -threshold 16 -word_size 7 | 0.93 | 106926.1 |
| -threshold 18 -word_size 3 | 0.90 | 106926.1 |
| -threshold 18 -word_size 4 | 0.88 | 12434.9 |
| -threshold 18 -word_size 5 | 0.92 | 12434.9 |
| -threshold 18 -word_size 6 | 0.93 | 47378.5 |
| -threshold 18 -word_size 7 | 0.92 | 42263.1 |
| -threshold 20 -word_size 3 | 0.90 | 22920.8 |
| -threshold 20 -word_size 4 | 0.82 | 6610.6 |
| -threshold 20 -word_size 5 | 0.89 | 13458.9 |
| -threshold 20 -word_size 6 | 0.92 | 22860.9 |
| -threshold 20 -word_size 7 | 0.90 | 22241.0 |

We changed –threshold, which is the threshold for neighborhood words, to 12 (default), 14, 16, 18, 20, 22, and 24, and -word_size, which is the subsequence length for the seed search, to 3 (default), 4, 5, 6, and 7. The first, second, and third columns show the parameters, the accuracy, and the computation time. When the parameters are “-threshold 12 -word_size 6” and “-threshold 12 -word_size 7”, BLASTX required > 96 hours, and we were unable to measure the computing time under these conditions.
